# DEMENTIA FAMILY CAREGIVERS’ REASONS FOR PARTICIPATION IN RESEARCH

**DOI:** 10.1093/geroni/igad104.1976

**Published:** 2023-12-21

**Authors:** Jasmine Vickers, Vicki Winstead, Henrietta Armah, Carolyn Pickering

**Affiliations:** University of Alabama at Birmingham, Birmingham, Alabama, United States; University of Alabama at Birmingham, Birmingham, Alabama, United States; University of Alabama at Birmingham, Birmingham, Alabama, United States; University of Alabama at Birmingham, Birmingham, Alabama, United States

## Abstract

Recruitment of participants with Alzheimer’s and Related Dementias (ADRD) and their family caregivers can pose significant challenges to the successful completion of research, particularly for intensive protocols like daily diary research. Understanding the reasons ADRD family caregivers are willing to participate in research can provide researchers with the tools for successful recruitment efforts. In this study, we assessed the reasons ADRD family caregivers (N=123) participated in a 21-day diary study and the translatability of the findings for informing the development of recruitment materials to address recruitment challenges. We conducted a thematic analysis of responses to the question “Tell us in a few words why you are interested in participating in this study” as an addendum to the consent form. Thematic analysis was conducted by two independent coders. Participants were mainly women (86%) and biological children of the care recipient (46%). Findings revealed four predominate motivations for participation in the research project: 1) finding meaning for their caregiving experiences by sharing them through the study, 2) altruistic motivations for helping others, 3) personal benefits including helping their care recipient or learning more about caregiving, 4) and the meaningfulness of the study’s focus. Caregivers were motivated to participate in this intensive protocol for a variety of meaning-making reasons. Recruitment materials should focus on language that emphasizes opportunities for caregivers to share their experiences and practical relevance of potential study findings.

